# Tumor-Microenvironment- Responsive Size-Shrinkable Drug-Delivery Nanosystems for Deepened Penetration Into Tumors

**DOI:** 10.3389/fmolb.2020.576420

**Published:** 2020-11-27

**Authors:** Xiaoliang Cheng, Houli Li, Xuemei Ge, Lijuan Chen, Yao Liu, Wenwei Mao, Bo Zhao, Wei-En Yuan

**Affiliations:** ^1^Department of Pharmacy, The First Affiliated Hospital of Xi’an Jiaotong University, Xi’an, China; ^2^Department of Food Science and Technology, College of Light Industry Science and Engineering, Nanjing Forestry University, Nanjing, China; ^3^Instrumental Analysis Center, Shanghai Jiao Tong University, Shanghai, China; ^4^Engineering Research Center of Cell and Therapeutic Antibody, Ministry of Education, and School of Pharmacy, Shanghai Jiao Tong University, Shanghai, China

**Keywords:** tumor, microenvironment responsive, size-shrinkable, drug delivery, nanosystems

## Abstract

Over the years, the manipulation and clinical application of drug-delivery nanosystems for cancer diseases have attracted a rapid growth of academic research interests, and some nanodrugs have been approved for clinic application. Although encouraging achievements have been made, the potency of nanomedicines in cancer treatment is far from satisfaction, and one significant reason is the inefficient penetration of nanoparticles into solid tumors. Particle size is one of the most significant features that influence diffusion ability of the drug-delivery system in tumors. Size-shrinkable drug-delivery nanosystems possess a size-switchable property that can achieve passive targeting *via* the enhanced permeability and retention (EPR) effect and transform into ultrasmall particles in tumors for deep penetration into tumors. The tumor microenvironment is characterized by acidic pH, hypoxia, upregulated levels of enzymes, and a redox environment. In this review, we summarize and analyze the current research progresses and challenges in tumor microenvironment responsive size-shrinkable drug-delivery nanosystems. We further expect to present some meaningful proposals and enlightenments on promoting deep penetration into tumors of nanoparticles.

## Introduction

Cancer is one of many major causes for mortality worldwide. Chemotherapy is a clinically practiced approach for treating cancer. In the past decades, the manipulation and clinical application of nanosized drug-delivery systems for the delivery of therapeutic and diagnostic payload for cancer diseases have attracted a rapid growth of academic research interests. Nanoparticles with sizes ranging from 1 to 100 nm have been confirmed the enhanced efficacy against cancers (Luo Q. et al., 2016; Kou et al., 2018a,b; He J. et al., 2019), and Doxil, Abraxane, and Genexol-PM have been approved for clinic treatment of cancers. Currently, organic and inorganic nanoparticles including liposomes, micelles, dendrimers, gold nanoparticles, lipid nanoparticles, albumin, magnetic nanoparticles, quantum dots, graphenes, and graphene oxides proceed to flourish in nanomedicine laboratories all over the world. Nanoparticles can accumulate and retain in tumors from circulating blood with leaky blood vasculatures, this process is referred to as enhanced permeability and retention (EPR) effect. In addition, tumor-specific ligands or antibodies, endogenous stimuli [e.g., acidic pH (Choi et al., 2020), hypoxia (Deng et al., 2018), enzymes highly expressed in tumors (Xiang et al., 2013), redox status (Sun et al., 2018), high concentration of glutathione/reactive oxygen species (ROS) (El-Sawy et al., 2018)] and external stimuli [e.g., temperature (Al-Ahmady et al., 2018), light (Zhang et al., 2018), magnetic field (Bocanegra Gondan et al., 2018), and ultrasound (Dwivedi et al., 2020)] were utilized to facilitate nanoparticles to achieve active tumor targeting. Although encouraging achievements have been made in tumor-targeting drug-delivery nanomedicines in recent years, the efficacy of nanomedicines in cancer treatment is far from satisfaction. Cancer cells can’t be effectively scavenged by nanodrugs leading to recurrence and metastasis of cancers, and the overall survival for patients has not been significantly improved in many cases, and one explanation for the phenomenon is the inefficient penetration of nanoparticles into solid tumors (Barenholz, 2012; Niu et al., 2018).

Solid tumors are characterized by a high density of extracellular matrix, elevated interstitial fluid pressure, and abnormal vasculature, as well as impaired lymphatic drainage. These unique histology characteristics constitute huge obstacles for nanodrugs to penetrate into the tumor, especially its core area and spatial diffusion through tumor (Yang et al., 2018), leading to failure in effective delivery of nanoparticles into the tumor far away from vasculature and weakened antitumor potency. On the other hand, size is one of the most significant features that influences diffusion ability of the drug-delivery system in the tumor, owing to the distribution distance that is inversely proportional to the diameter of nanoparticles (Huang et al., 2012). Although larger nanoparticles with diameters of approximately 100–150 nm possess the advantages of passive tumor targeting *via* the EPR effect, improved pharmacokinetics profile, and prolonged blood circulation, they are inferior in deep diffusion in tumors due to huge distribution obstacle (Hu et al., 2018c; Yang et al., 2019). On the contrary, ultrasmall size nanoparticles of below 20 nm (Huo M. et al., 2017) or 10 nm (Yang et al., 2019) exhibit relatively higher penetration capability and interstitial transport. However, ultrasmall nanodrugs are rapidly eliminated from circulating blood through renal filtration, resulting in ineffective tumor accumulation (Hu et al., 2018a). To solve the dilemma, an ideal drug-delivery vehicle should possess a size-switchable property that is of large diameter in systemic circulation to achieve passive targeting *via* the EPR effect and transform into ultrasmall particles in tumors by stimulus to deeply penetrate into tumor.

Compared to normal tissue, the tumor microenvironment shows unique properties, such as acidic pH, upregulated certain enzymes, hypoxia, redox environment, and ROS. The tumor microenvironment responsive drug-delivery systems utilize the histology characteristics of tumors, thus realizing an effective approach for site-specific release of therapeutic and diagnostic drugs. Unlike external physical stimuli such as light and ultrasonic, endogenous stimuli are readily available, and no additional instrument and extracorporeal stimulus are needed. In this review, we summarize and analyze the current research progresses and challenges in the tumor microenvironment responsive size-shrinkable drug-delivery systems, especially many novel multistrategy approaches based on the tumor microenvironment response conjugated with other stimulus are discussed.

## Acidic pH Responsive Size Switchable Nanovehicles

Due to the biological environment of tumor tissue of relative low pH value, pH-responsible linkage could be designed and incorporated into the nanoparticle for the purpose of formulating a size-shrinkable drug-delivery system. It can provide a new and effective modality for tumor-targeting delivery, and several types of these nanoparticles were developed.

One method for improving the targeting delivery efficiency of nanoparticles for cancer therapy is to develop nanovesicles with changeable sizes and surface characteristics, such as Zeta potential, poly(ethylene glycol) (PEG) shielding or deshielding, and conjugating of different targeting moieties to reach the desired targets. Chen and colleagues designed size-shrinkable nanoparticles with core-shell structure by electrostatic interaction. Dimethylmaleic anhydride-modified methoxy poly(ethylene glycol)-*block*-poly(L-lysine) as the shell of the nanoparticle was negatively charged with a pH-sensitive bond, and the core was positively charged with a disulfide cross-linked polypeptide. The nanoparticles underwent remarkable size reduction from approximately 145 to 40 nm, and surface charge reversed from negative to positive at an acidic tumor microenvironment. The nanoparticles could penetrate about four times deeper than that of the non-transformable one, and almost eradicated the xenografted carcinoma in mice (Chen et al., 2017).

Chemotherapy is one of the effective strategies to fight against cancer. However, multidrug resistance may pump these drugs such as doxorubicin (DOX) out of the target cells and thus reduce their therapeutic efficiency (Bock and Lengauer, 2012; Flemming, 2015; Liu et al., 2017). To overcome this unwanted effect, some nanoparticles were designed to deliver these therapeutic drugs to the target sites directly. It is reported that most of anti-cancer drugs such as DOX need to be released in nucleus to induce nuclear DNA damage. Diameter is the key for optimizing these delivery systems to tailor the size of nanoparticle large enough to accumulate in tumor tissues and with the right size to pass nucleopores, which was reported as 39 nm in diameters to entry the nuclear and release drugs (Pante and Kann, 2002). One size-changeable polymer micelle was reported to solve this problem. Poly(ethylene glycol)-polylactide-ss-polyethylenimine-2,3-dimethylmaleic anhydride (mPEG-PLA-ss-PEI-DMMA) was synthesized to form a micelle. PEG block was used to shield the positive charges of polyethylenimine (PEI) and prolong the circulation time *in vivo*. PEI functions as a pH-responsible block to enlarge size change when accumulated in biological acidity tumor tissues by EPR effects, and it also facilities endosome escape by the proton sponge effect (Zhu and Mahato, 2010; Guo et al., 2015; Lee et al., 2017; Vermeulen et al., 2018). From the results, it can be concluded that the particle sizes increased from 42.1 nm to 87.9 nm with the decrease of pH values. The degradation of disulfide bonds in appearance of intracellular glutathione may remove the PEI block and thus produce the relative smaller-sized poly(ethylene glycol)-poly(-caprolactone) (PEG-PCL) with optimized molar ratio to form micelles to go across the nucleopores and release its payloads (Guo et al., 2015).

For deep tumor penetration, a reversible swell-shrinking nanogel was used as a nanoparticle tailor for a desired size. It consists of N-lysinal-N′-succinyl chitosan with an isoelectric point around 6.0 to offer an acid-triggered charge reversal capability, poly(N-isopropylacrylamide) and negatively charged macromolecule bovine serum albumin. This structure can provide the possibility of the pH-responsive swelling and shrinking process, which may keep the nanoparticle stable in pH 7.4, swelling in the endosome for rapid escape from endosomal with pH value of 4.0 to 5.0, shrinking back to its original in cytosol with pH 6.8 to 7.4, to repeat the process for neighboring diseased cells (Ju et al., 2014).

## Hypoxia-Responsive Size-Shrinkable Nanodrugs

**FIGURE 1:**
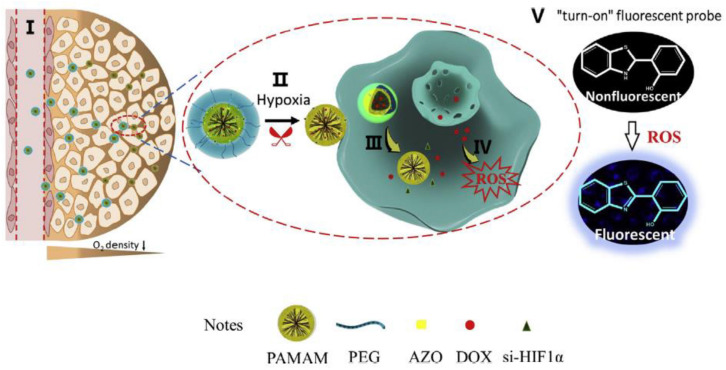
Schematic illustration of the transport path for a hypoxia responsive size-shrinkable nanoparticle PEG-AZO-PAMAM (PAP) for co-delivery of doxorubicin, siRNA, and a ROS probe to increase penetration into tumor. **(I)** Blood circulation: PAP was efficiently transported to the tumor site *via* long circulation time of PEG in blood and the EPR effects. **(II)** Tumor microenvironment: AZO was degraded in hypoxic tumor microenvironments, and PEG was removed to realize size shrinkage and expose positively charged PAMAM that help to penetrate into the tumor core. **(III)** Within the tumor cells: Through the proton pump effect, PAMAM escaped from endosomes to release DOX and siRNA. **(IV)** An increased level of ROS induced by DOX. **(V)** Fluorescence imaging: The elevated ROS level induced by DOX could trigger the “turn-on” of fluorescent probe (reproduced with permission from Xie et al. (2018). Copyright 2018 Elsevier).

Hypoxia is a hallmark feature of the tumor microenvironment resulted from an imbalance between overwhelming consumption of nutrient and oxygen by rapidly growing cancer cells and an inadequate supply of oxygen by the aberrant angiogenesis and impaired blood vessels (Saikolappan et al., 2019). The oxygen partial pressure (pO_2_) decreases from vasculature to the core of the tumor, and comparing with 46–76 mmHg pO_2_ in healthy tissues hypoxia area with pO_2_ of below 10 mmHg is created (Yao et al., 2018). Due to the significant role of hypoxia in cancer multidrug resistance, angiogenesis, invasion, and metastasis (Wilson and Hay, 2011), persistent efforts have been put forward to develop a targeting hypoxia region or hypoxia responsive nanoparticles. Xie et al. fabricated a hypoxia-responsive size-shrinkable nanoparticle for co-delivery of DOX, siRNA, and a ROS probe to increase penetration into the tumor (Xie et al., 2018). The size-switchable nanovehicle was designed by conjugation of the polyamidoamine (PAMAM) dendrimer, which was a globular-shaped macromolecule with an ultrasmall size to PEG 2000 *via* a hypoxia-sensitive linker azobenzene (AZO) (Figure 1). The DOX and probe were loaded into the hydrophobic core of PAMAM, and a hypoxia-inducible factor 1α (HIF-1α) siRNA was bound to the periphery of the PAMAM dendrimer *via* electrostatic interactions between anionic siRNA and amine groups on the surface of PAMAM. Once reaching the hypoxic microenvironment, the PEG was cleaved from the PAMAM surface due to the degradation of AZO to aminoaromatics, resulting in the emancipation of ultrasmall-size PAMAM of 5.4 nm and deep penetration of the payloads.

## Enzymes-Responsive Size-Changeable Nanodrugs

The tumor microenvironment expresses upregulated levels of enzymes in many kinds of tumors, such as matrix metalloproteinase (MMP) and hyaluronidase (Ding et al., 2014; Hu et al., 2014). A tumor-associated enzyme-triggered drug release is one of the most specific and potent strategies to realize effective delivery of drugs to tumors.

### Matrix Metalloproteinases-2 Triggered Size Reduction

Matrix metalloproteinase-2 (MMP-2), a family of proteolytic enzymes, exhibits a critical role in carcinoma angiogenesis, progression, metastasis, and invasion through degrading structural components of the extracellular matrix (Egeblad and Werb, 2002; Kessenbrock et al., 2010). MMP-2, which is secreted by cancer cells and tumor stromal cells, is considered as a biomarker in many types and grade of cancers (Stankovic et al., 2010; Gialeli et al., 2011; Zhu and Torchilin, 2013), and MMP-2 has been generally accepted as a target for active targeting for tumors. Several strategies have been proposed to fabricate MMP-2-responsive size-shrinkable nanoparticles.

**FIGURE 2:**
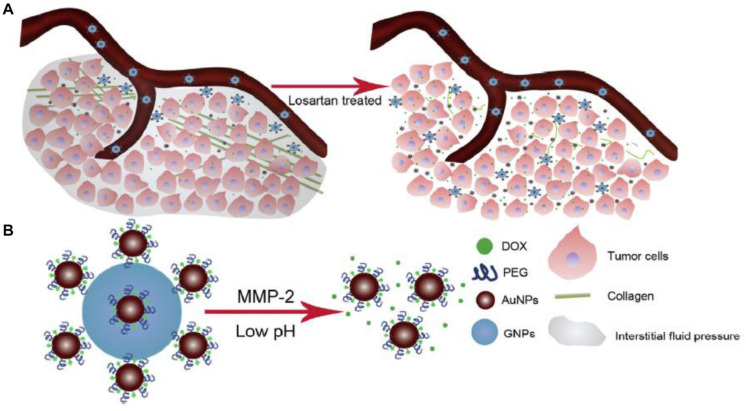
Elucidation of the dual strategy of combination of size shrinkable nanoparticles with collagen depletion by losartan. **(A)** Losartan treatment reduced the collagen level of xenografted tumor, leading to deeper penetration of DOX-loaded nanogold onto gelatin nanoparticles. **(B)** MMP-2-triggered size shrunk and low-pH-induced DOX release in tumor (reproduced with permission from Cun et al. (2016). Copyright 2016 Elsevier).

Gelatin, a most extensively used natural polymer, is the substrate of MMP-2 (Akkoc et al., 2017), and degradation of gelatin nanoparticles by MMP-2 is applied in design of MMP-2 sensitive-size tunable nanovehicles. The surface-carrying strategy is tethering ultrasmall nanoparticles to the periphery of large nanoparticles to form a nanocomplex with raspberry-like structure (Li J. et al., 2015; Ruan et al., 2015a; Huo M. et al., 2017). Gelatin nanoparticles with size of about 150–200 nm (Madkhali et al., 2019), and surface-carrying strategy, is applied to link small nanovehicles, for example, gold nanoparticles, quantum dots, and dendrimers to the surface of gelatin nanoparticles. Cun et al. (2016) developed a size-shrinkable nanoparticle through fabricating DOX-loaded nanogold onto gelatin nanoparticles *via* PEG (Figure 2), and nanocomplex decreased from more than 117.8 nm to less than 50.0 nm and released gold nanoparticles under the stimulus of MMP-2. However, the drug-loaded nanogold with size of approximately 50 nm is to large for potent deep penetration into the tumor. In order to enhance tumor penetration, the tumor was pretreated with losartan to deplete tumor collagen, which was the main ingredient of the tumor extracellular matrix. The size-changeable nanovehicles showed striking tumor penetration efficiency and tumor-inhibition potency. Many types of MMP-2-triggered gelatin nanoparticles carrying nanogold on its periphery were reported (Cun et al., 2015; Ruan et al., 2015a,b). Hu et al. (2015) connected arginine-glycine-aspartic acid peptide-conjugated dendritic poly-L-lysine loaded with DOX to gelatin nanoparticles to establish an MMP-2-sensitive size-shrinkable drug delivery system. This multistage nanovehicle shrank from 200 to 30 nm and showed higher tumor retention and deeper penetration than gelatin nanoparticles or dendrimers. An MMP-2-sensitive nanoparticle with a core composed of gelatin and a surface covered with quantum dots was engineered, the core of 100 nm gelatin nanoparticles was degraded, and 10-nm quantum dots were released from their surface (Wong et al., 2011). The multistage nanovehicles demonstrated both the long circulating half-life, which was necessary for EPR effect, and deep tumor penetration into a dense collagen matrix.

**FIGURE 3:**
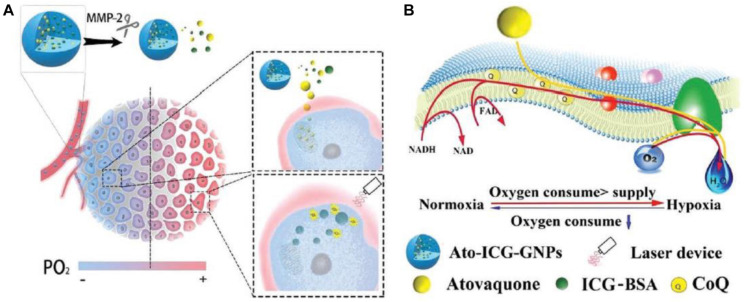
**(A)** Schematic illustration of atovaquone (Ato) and indocyanine greenbovine (ICG) serum albumin nanocomplex encapsulated in gelatin nanoparticle for enhancing the tumoricidal effect exerted by photodynamic therapy treatment. Once entered into tumor, the gelatin nanoparticle was ruptured with the assistance of MMP-2, releasing ICG serum albumin nanocomplex and Ato. **(B)** A schematic showing the broad influence of Ato on oxidative phosphorylation, and the mechanism underlying the reverse of hypoxia as assisted by Ato and ICG serum albumin nanocomplex encapsulated in gelatin nanoparticle (reproduced with permission from Xia et al. (2019). Copyright 2019 John Wiley and Sons).

The Trojan horse strategy—hiding small nanoparticles, referred to as Greek soldiers inside the large nanoparticles and releasing of small nanoparticles under certain trigger—was also utilized in fabrication of MMP-sensitive size-tunable nanoparticles. Xia et al. (2019) developed a size-shrinkable gelatin-based vehicle in which the bovine serum albumin nanocomplex was encapsulated in gelatin nanoparticles for photodynamic therapy (Figure 3). Upon cleavage, due to the presence of MMP-2, the released small-size vehicles delivered drugs deeply into tumor hypoxic region. Paclitaxel loaded and Pluronic^®^ F127-modified porous hollow magnetic subnanocarriers were further assembled through gelatin conjugation to form a core-shell structure with multiple subnanocarriers entrapped in a gelatin matrix, and the core-shell non-vehicles were enzymatically degraded from about 140–160 nm to ∼20 nm by MMP-2 (Lai et al., 2018). Losartan was loaded in gelatin nanoparticles to decrease collagen in extracellular matrix, and magnetic nanoparticles liberated from core-shell nanovehicles showed deeper penetration. Small polyamidoamine (PAMAM) dendrimers (∼5 nm) were encapsulated in large gelatin nanoparticles (∼200 nm), the multistage nanocarrier was stable during systemic circulation, and PAMAM dendrimers were released in response to high MMP-2 enzymes in tumor microenvironment (Fan et al., 2017). This multistage nanovehicle exhibited great potential in improving anticancer efficacy.

**FIGURE 4:**
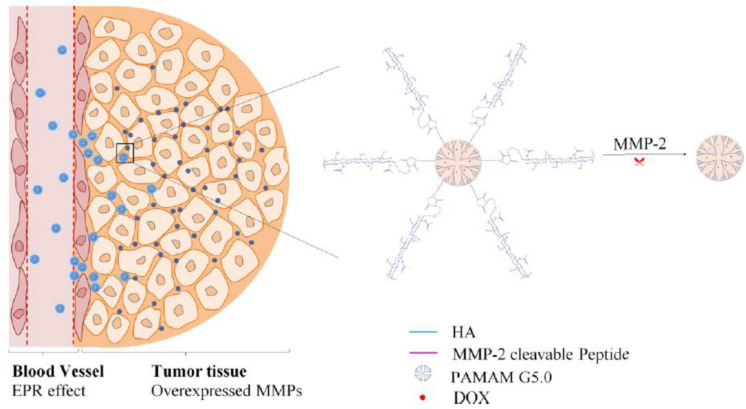
Schematic illustration of size shrinkage of the HA-PLGLAG-PAMAM from 200 to 10 nm triggered by MMP-2, a protease highly expressed in the tumor extracellular matrix, thus achieving deep penetration into tumor and improved therapeutic efficacy (reproduced with permission from Han et al. (2017). Copyright 2017 American Chemical Society).

The “peeling onions” strategy is that the outer shell layer of nanoparticles is cleaved in response to external of internal stimuli and is an approach for the design of size-tunable nanovehicles. Conjugation of the terminal glucose of hyaluronic acid to the amidogen on PAMAM surface *via* an MMP-2-responsive peptide (PLGLAG) to form MMP-2-sensitive size-shrinkable nanovehicles (Han et al., 2017). The nanoparticles experienced a dramatic and quick size shrink from an initial size of ∼200 nm to ∼10 nm because of cleavage of PLGLAG in the presence of MMP-2 (Figure 4). The nanoparticles achieved fast diffusion, deep penetration, and improved therapeutic efficacy.

Clusters of ligands targeting the chemokine (C-C motif) ligand 28 (CCL28)-modified tungsten-oxide nanoparticles were covalently bound *via* an MMP-2-cleavable peptide Pro-Leu-Gly-Val-Arg-Gly (Huo D. et al., 2017). The half-life of a cluster of tungsten-oxide nanoparticles in blood was increased compared to that of tungsten-oxide nanoparticles because of the enlarged size of approximately 33 nm. Once the clusters of nanoparticles accumulated inside the tumor due to the EPR effect, upregulated content of MMP-2 in the tumor microenvironment destructed the clusters to release small nanoparticles (∼5 nm), which deeply penetrated into the hypoxia region of the tumor.

### Hyaluronidase

Hyaluronic acid (HA), a natural non-sulfated glycosaminoglycan, consists of alternating units of D-glucuronic acid and N-acetyl-D-glucosamine connected through β-1,3- and β-1,4-glycosidic bonds (Dosio et al., 2016). HA is the content of many human organs such as body fluids, the extracellular matrix, and connective tissues (Vizoso et al., 2004; Yin et al., 2006). Due to its biocompatibility, biodegradability, non-toxicity, and non-immunogenicity properties and its overexpressed receptor cluster of differentiation (CD) protein CD44 on many tumor cells, HA is widely applied in antitumor drug, deoxyribonucleic acid (DNA), and siRNA delivery (Luo et al., 2019). Hyaluronidases, the specific enzymes for degradation of HA, are demonstrated to be associated with tumor progress (McAtee et al., 2014) and 20–1,000 times higher in many cancers than in health organs (Lokeshwar et al., 2001; Benitez et al., 2011). Scientists utilized the high content of hyaluronidase in the tumor microenvironment to design hyaluronidase-responsive size-changeable nanovehicles.

**FIGURE 5:**
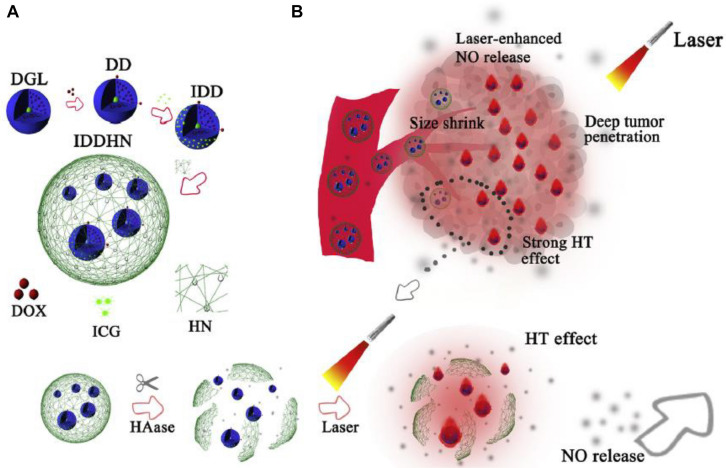
**(A)** Schematic design of hyaluronidase-triggered size-shrinkable HA shells, which were modified with NIR laser-sensitive NO donor (HN), small-sized dendrimeric prodrug (IDD) of DOX as chemotherapy agent and indocyanine green (ICG) as photothermal agent into a single nanoparticle. **(B)** Synergistic effects for deep tumor penetration and therapy effects were realized *via* rupture of HA shells triggered by hyaluronidase and improved EPR effect by laser-enhanced NO release upon strong hyperthermia effect of indocyanine green (reproduced with permission from Hu et al. (2018b). Copyright 2018 Elsevier).

Huo et al. encapsulated PAMAM dendrimers into HA nanoparticles using the Trojan horse strategy. The HA/PAMAM nanosystems with a large scale of about 197 nm were stable during systematic circulation, and once reaching the tumor site, they were degraded by the highly expressed hyaluronidase. PAMAM dendrimers with a small size of 5.77 nm and positive charge were released (Huo M. et al., 2017). A small-sized dendri-graft-L-lysine dendrimer conjugated with DOX and indocyanine were wrapped into nitric oxide (NO) donor-modified hyaluronic acid nanoparticles, and the multistage-responsive nanoparticles could be rapidly degraded from approximately 330 nm to smaller sizes, most of which were at 35–60 nm after incubation with hyaluronidase (Hu et al., 2018c). Hu et al. (2018b) engineered an intelligent nanoparticle with dendrigraft poly-L-lysine dendrimer loaded DOX and photothermal agent indocyanine green as core and near-infrared (INR) laser-sensitive NO-donor-modified HA shells. Synergistic deep penetration was achieved through degradation of HA shells by hyaluronidase and improved EPR effect by laser-enhanced NO release upon strong hyperthermia effect of indocyanine green (Figure 5). Liu et al. (2018) employed HA and cationic bovine serum albumin-protected gold nanocluster to successfully construct size-reducible nanoplatform, and 200 nm of the nanovehicle with optimal EPR effect was screened out for further loading drug for chemo-photothermal therapy. Series of hyaluronidase triggered size-reducible HA-coated cationized gold nanoclusters, which was further shielded with red blood cell membranes in different initial diameters, were synthesized, and the size-reducible nanoparticles could be hydrolyzed into the small cores in the presence of hyaluronidase. The optimal initial size of 150 nm was filtered (Yu et al., 2019). A “cluster bomb” containing HA nanogels core loading DOX and transient receptor potential ankyrin 1 (TRPA-1) inhibitor and tumor homing peptide tLyp-1 (CGNKRTR) modified distearoyl phosphoethanolamine-(polyethylene glycol) 2,000 (DSPE-PEG2000) micelles, which were carried on the surface of nanogels was designed (Wang et al., 2020). The nanogels were cracked into HA fragments triggered by high hyaluronidase in the tumor microenvironment, and exposure of HA target and reduction of nanoparticle size were realized. Benefiting from the small particle diameter and targeting ability of HA and ligand modified on micelles, the HA fragments carrying micelles on periphery achieved deep penetration into the tumor.

## Redox-Environment-Responsive Size-Tunable Nanovehicles

### Glutathione-Triggered Size Reduction

The intracellular glutathione concentration in the tumor microenvironment is in the range of 1–10 mmol/L, which is many times higher than that in the extracellular of healthy organs (Li Z.Y. et al., 2015; Wang et al., 2018). The significant difference in the reductive potential between cancer and normal tissues has been utilized as a promising strategy to achieve reduction responsive drug delivery into tumor (Luo C. et al., 2016; John et al., 2017). The disulfide bond is such a bioreducible linkage that is easy to degrade in the reductive potential environment (Chen et al., 2015; Uthaman et al., 2018).

**FIGURE 6:**
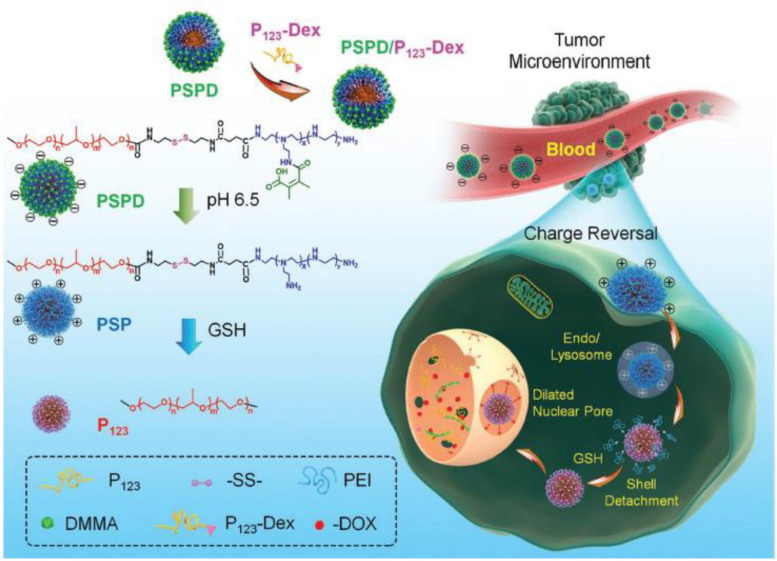
Schematic illustration of a size reducible core-corona nanostructure that was self-assembled by two unimers of conjugated Pluronic P_123_ to DMMA-PEI (PSPD) *via* disulfide bond and dexamethasone modified Pluronic P_123_ (P_123_-Dex). The core-corona nanostructure efficiently delivered DOX into the nucleus of tumor cells through exploiting acidic pH and intracellular redox potential, as well as the ability of dexamethasone to target and dilate nuclear pores (reproduced with permission from Wang et al. (2017). Copyright 2017 John Wiley and Sons).

Wang et al. conjugated amphiphilic blocks Pluronic P_123_ to charge-reversible blocks 2,3-dimethylmaleic anhydride (DMMA)-polyethylenimine (PEI) *via* the disulfide bond to fabricate a size-reducible hybrid micelle, and a dexamethasone-modified Pluronic P_123_ amphiphilic block was applied to target nuclei and dilate nuclear pores (Figure 6). The two unimers self-assembled into a core-corona nanostructure, the cleavage of disulfide group occurred quickly in glutathione-elevated cancer cells followed by size reduction due to detachment of polyethylenimine from the micelle corona (peeling onion strategy) (Wang et al., 2017). A novel multifunctional size-switchable nanovehicle based on carboxylic functionalized axial ligands of Pt(IV) complexes as linker to integrate ZnFe_2_O_4_ nanoparticles on the surface of upconversion nanoparticles was developed for synergistic cancer therapy including photodynamic therapy, chemotherapy, and Fenton reaction (Bi et al., 2018). The original size of the nanosystem was about 100 nm, which was beneficial for the EPR effect and tumor accumulation, and in the tumor microenvironment Pt(IV) prodrug as a linker was broken, owing to the reducing property of glutathione, switching to upconversion nanoparticles of 25 nm and ZnFe_2_O_4_ of 7 nm. Guo et al. (2015) applied a novel size-changeable polymer micelle to form a core-corona structure, and the two end of poly lactide as the core material was conjugated with methoxy polyethylene glycol and polyethylenimine through disulfide group, respectively. The two hydrophilic polymers were used as the corona material. The degradation of disulfide bonds as linker between poly lactide and polyethylenimine was triggered by intracellular glutathione, resulting in the deshielding of polyethylenimine corona and size reduction without demicellization. The smaller micelles were capable of access to the nucleus due to the reduced size (Guo et al., 2015).

### Reactive Oxygen Species

The level of ROS, including superoxides (O_2_^–^), hydroxyl radicals (OH^∙^), hydrogen peroxides (H_2_O_2_), and singlet oxygen (^1^O_2_), are approximately 100 times higher in cancer cells than that in normal cells because of their constant production as the byproducts by mitochondria in aerobic cells during energy production (Ngo et al., 2015; Mo and Gu, 2016). Based on the high content in cancer, many ROS responsive nanocarriers for site-specific drug delivery and release were reported. ROS-responsive groups, such as ferrocenyl, arylboronic ester, thioether, thioketal and selenium units, are commonly employed in development of ROS-stimulated nanovehicles (Sun et al., 2017; Luo et al., 2018). The ROS-sensitive linkers are degraded or switched from hydrophobic to hydrophilic in response to oxidization of ROS, resulting in nanocarriers intracellular disassembly and corresponding payload release (Saravanakumar et al., 2017; He Y. et al., 2019).

Cao et al. (2018) have developed ROS-responsive polymeric nanocarrier to realize remotely controlled drug release by light-activated size shrinkage. With the assistance of an amphiphilic copolymer poly(ethylene glycol)-b-poly(ε-caprolactone), a ROS-responsive poly(thioketal phosphoester) self-assembled to form a ROS-sensitive polymeric nanovehicle encapsulating chlorin e6 and DOX. Under the red-light irradiation, thioketal linker was cleaved by the ROS produced by encapsulated chlorin e6, resulting in rapid degradation of nanovehicle core and size shrinkage.

## Conclusion and Perspectives

Over the years, many significant progresses have been achieved in enhancing deep penetration of nanoparticles into tumors. Recent advances in the field of the tumor microenvironment triggered size-shrinkable drug-delivery nanosystem, which utilized the unique profiles of the tumor microenvironment such as low pH, hypoxia, upregulated expression of certain enzymes, redox species, and reactive oxygen levels are summarized in this review. However, great challenges are faced in the scientific research and especially in clinical application.

First, the heterogeneity of cancer is relatively complex and highly varied among different tumors, pathology, and clinical stages (Alizadeh et al., 2015; Singh et al., 2015). The influence factors on hampering penetration capability of nanoparticles are very complicated rather than only size dependence. Therefore, design of the tumor microenvironment stimulated size-switchable nanoparticles and combination with different treatment strategies should be based on further basic research in tumor biology, pathology, and clinical stage. Second, the biocompatibility, biodegradability, and safety for size-shrinkable nanoparticles and other multistage vehicles *in vivo* and *in vitro* should be carefully evaluated (Sanna et al., 2014; Hjorth et al., 2017). Last but not least, druggability also should be highly concerned in development of size-shrinkable nanoparticles. The pharmaceutical industry favors the “keep it simple, stupid” (KISS) principle (Crommelin et al., 2020). Manufacture techniques for complicated and smart size-shrinkable nanoparticles are difficult to scale up from laboratory scale to industry scale. In 2019, Clinicaltrials.gov exhibited no trial in progress, which is searched for the disease “cancer” and the other term “size-shrinkable nanoparticle.” Aiming at improving the prospect of clinical application, size-tunable nanosystem with simple structure should be developed.

In conclusion, there are great challenges for treatment of cancer, and the strategy of tumor-microenvironment-responsive size-changeable nanovehicles have demonstrated encouraging achievements in scientific research fields of promoting penetration into tumors of nanoparticles. We look forward that more simple size-shrinkable nanocarriers based on further basic research in tumor biology, pathology, and clinical stage are developed and translated into clinic in the recent future, and more patients benefit from nanotechnology.

## Author Contributions

YL, WM, BZ, and W-EY conceived the overall idea and concept and revised the manuscript. XC, HL, and XG drafted and edited the manuscript. LC drew the figures. All authors reviewed and approved the final version of the manuscript.

## Conflict of Interest

The authors declare that the research was conducted in the absence of any commercial or financial relationships that could be construed as a potential conflict of interest.
